# Dynamics of Perceived Stress, Stress Appraisal, and Coping Strategies in an Evolving Educational Landscape

**DOI:** 10.3390/bs14070532

**Published:** 2024-06-25

**Authors:** Aneela Maqsood, Seema Gul, Nazia Noureen, Arooj Yaswi

**Affiliations:** 1 National Centre for Research on Suicide Prevention, Department of Behavioral Science, Fatima Jinnah Women University, Rawalpindi 44000, Punjab, Pakistan; 2 General Studies Department, College of Humanities and Sciences, Prince Sultan University, Riyadh 11586, Saudi Arabia; sgul@psu.edu.sa (S.G.); ayaswi@psu.edu.sa (A.Y.); 3 Department of Psychology, Foundation University, Rawalpindi 44000, Punjab, Pakistan; nazia.ch88@gmail.com

**Keywords:** perceived stress, stress appraisal, college teachers, coping

## Abstract

In the evolving landscape of education, college teachers often find themselves at the crossroads of myriad stressors, ranging from institutional demands to personal challenges. Understanding the factors that influence their stress perceptions and coping mechanisms is pivotal, not just for their well-being, but also for the overall quality of education imparted. This study sought to investigate the intricate relationships between gender, marital status, institutional affiliation, and their collective impacts on perceived stress, stress appraisal, and coping strategies among college teachers. A sample of 300 college teachers, evenly split with reference to gender, was engaged. Employing tools including Perceived Stress Scale (PSS), Stress Appraisal Measure (SAM), and Brief COPE, the analysis of the study used independent samples *t*-test and Pearson Product Moment Correlation to derive insights. Findings revealed pronounced gender disparities in stress perception and appraisal, with women teachers manifesting heightened levels. Marital status emerged as a significant modulator of stress and coping, with married teachers depicting nuanced stress and its appraisal with reported coping strategies compared to their unmarried peers. Furthermore, a significant variance in perceived stress and coping was observed between teachers of private and governmental institutions, with private institution teachers displaying elevated stress levels. The study underscores the multifaceted nature of stress experiences among college teachers in Pakistan, influenced by a blend of personal and institutional determinants. These insights are pivotal for institutions aiming to enhance the well-being and effectiveness of their teachers. However, given the specific cultural context of the study, there is an imperative for more global, comparative research, ensuring holistic support mechanisms for teachers worldwide.

## 1. Introduction

In an era characterized by rapid technological advancements, globalization, and increased demands in the workplace, the professional landscape has undergone significant transformations [1]. The implications of these changes on individual psychological health are profound, with professionals across sectors facing the brunt of increased expectations, performance pressures, and a myriad of stressors [2,3]. Within the nexus of professional life, stress has emerged not merely as a random inconvenience but as a pervasive element that can potentially shape career trajectories, work cultures, and personal well-being [4].

Stress, inherently, is not a toxic phenomenon [5]. In evolutionary terms, it equipped humans with mechanisms to respond effectively to immediate threats [5]. However, in contemporary settings, where threats are more psychological than physical, stress has taken on a chronic character, influencing cognitive appraisals, emotional responses, and behavioral tendencies [2]. Stress appraisal is a fundamental cognitive process that plays a key role in determining how an individual perceives, interprets, and subsequently responds to stressors [6]. This represents the individual’s evaluation of what related to him or her is at stake and what can be done about it. These appraisals are instrumental in dictating the coping mechanisms an individual might deploy to counteract or manage the stressors [6].

Coping, in its essence, can be understood as the cognitive and behavioral strategies that individuals use to manage taxing internal and external demands [7]. These strategies can vary significantly across individuals and situations, ranging from problem-focused approaches, where individuals target the root cause of stress, to emotion-focused strategies, where individuals attempt to regulate the emotional fallout of the stressor [8]. The efficacy of these coping strategies is not just crucial for individual well-being but also has broader ramifications for organizational health, team dynamics, and overall productivity [9].

The teaching profession, universally, is considered to be demanding and potentially stressful [10]. In the context of Pakistan, a study by Maqsood et al. [11] posited a similar proposition and suggested the need for policy reforms for teachers to improve the quality of their work life [11]. Over the past few decades, the role of teachers has expanded significantly to accommodate societal changes, evolving pedagogical methods, and greater administrative burden [10]. This ever-increasing set of expectations and responsibilities has led to heightened levels of perceived stress among teachers across different cultural contexts [12].

Educational institutions, often perceived as bastions of knowledge and personal growth, are not immune to the influences of these societal shifts [13]. Teachers, the linchpins of the educational ecosystem, are finding themselves navigating a landscape replete with challenges [14]. Beyond the traditional demands of pedagogy, they grapple with increased administrative burdens, technological integrations, student mental health issues, societal expectations, and in many regions, economic and political upheavals [7]. Especially in contexts like Pakistan, where socio-political dynamics, including terrorism, unstable governance, and economic uncertainties, exert additional pressures, the stress experienced by teachers—who are pivotal in shaping the future of the nation—can be acute.

While teachers globally face challenges such as curriculum changes, increased workloads, and administrative pressures, Pakistani college teachers additionally grapple with security concerns, inconsistent governmental policies on education, and an environment of socio-political unrest. These elements can intensify their perceived stress and the orientation to cope more or less with the stressors [15].

### Gender, Marital Status, and Institutional Affiliation

Diverse demographic factors such as gender, marital status, and institutional affiliation also significantly influence perceived stress and coping mechanisms among teachers [16,17]. Historically, research has shown that women often report higher levels of perceived stress compared to men [17,18]. Additionally, the dichotomy of Private vs. Government institutions can result in varied working conditions, expectations, and support structures, leading to differential stress perceptions among teachers in these settings [3,19].

The significance of marital status is yet another dimension worth exploring. The combined pressures of professional responsibilities and familial obligations can have a cumulative effect on perceived stress levels, especially in cultures where familial responsibilities are deeply entrenched, as is the case in Pakistan [16,20].

Understanding the unique interplay of these factors is imperative for formulating effective interventions aimed at enhancing teachers’ well-being and professional efficacy. While there is a vast body of research on teacher stress and coping mechanisms in various countries, a deep dive into the context of Pakistani college teachers, with the aforementioned nuances, remains a gap in the literature. This study, therefore, seeks to address this lacuna, aiming to provide valuable insights into perceived stress, appraisal, and coping strategies among college teachers in Pakistan.

Although teachers around the world encounter multifaceted challenges, the situation in Pakistan presents a distinctive case worthy of examination. This research aims to uncover the relationship between perceived stress, stress appraisal, and coping mechanisms within the cultural, socio-political, and environmental context of Pakistan. This study is not just about understanding stress but also about acknowledging and appreciating the resilience and adaptability of teachers in challenging circumstances.

## 2. Literature Review

### 2.1. Perceived Stress

In today’s fast-paced world, coupled with the mounting pressures of professional and personal challenges, stress has become a major issue affecting people from all walks of life [2]. At its essence, stress is a phenomenon that is influenced by how individuals perceive and assess their surroundings [21]. Lazarus and Folkman [22] articulate stress as “*a transactional relationship between the person and the environment appraised by the person as taxing or exceeding his or her resources and endangering his or her well-being*” (p. 33). This definition accentuates the dynamic interplay between individuals and their environment, emphasizing the subjective nature of stress perception.

Perceived stress, in essence, underscores how one understands the challenges posed by their environment in relation to their capacity to respond. It is not merely about the existence of stressors but also an individual’s assessment of their magnitude and implications. Such perceptions, influenced by a myriad of internal and external factors, can subsequently dictate emotional, cognitive, and physiological responses [2,23].

### 2.2. Perceived Stress among Teachers

The realm of academia, with its unique demands and pressures, presents a distinctive set of stressors. Teachers, as the primary actors in this context, often find themselves at the crossroads of institutional demands, societal expectations, student needs, and personal aspirations [10]. Given the multifarious roles they play—as teachers, mentors, counselors, and administrators—teachers are inevitably exposed to a plethora of challenges that can exacerbate their stress perceptions.

Recent literature has delved into the nuanced manifestations of perceived stress among teachers. For instance, an exploration of teachers in urban environments highlighted the acute stress stemming from administrative pressures, lack of resources, and classroom management challenges [24]. Their findings resonate with those who elucidated how curriculum changes, student behavior, and inadequate professional development opportunities heighten stress perceptions among teachers [7].

Furthermore, the emotional labor inherent in teaching—where teachers often suppress or amplify emotions to conform to professional norms—can intensify perceived stress. As a note, the emotional regulation required in managing diverse classrooms can lead to burnout, reduced job satisfaction, and elevated stress levels [25].

Teachers’ perceptions of stress are also shaped by their environments. For instance, in regions grappling with socio-political upheavals or economic challenges, teachers may perceive heightened stress due to safety concerns, job instability, or diminished resources. The authors of [24,26] in their study on teachers underscored the amplified stress perceptions arising from socio-political instabilities and their implications on teaching efficacy.

In short, perceived stress, especially among teachers, is a multifaceted construct shaped by individual appraisals, job demands, marital status, institutional factors, and broader socio-cultural contexts. Recognizing and addressing these stress perceptions is pivotal given the centrality of teachers in shaping future generations.

### 2.3. Stress Appraisal

Human reactions to stressors are not just mechanical responses but are deeply rooted in cognitive processes that assess the significance and implications of such stressors. This evaluation, termed as “stress appraisal”, forms the crux of how an individual perceives, interprets, and ultimately responds to challenging situations. Central to this concept is the understanding introduced by Folkman [21], who described cognitive appraisal as a person’s assessment of whether an event or situation is detrimental to their well-being, and also the options available to manage the event or cope with the issue.

The process of appraisal is broadly divided into primary and secondary stages. The primary appraisal, as delineated by Folkman et al. [21], represents an individual’s initial assessment of the potential threat posed by a stimulus. This foundational evaluation discerns if the situation at hand is detrimental to one’s well-being. Within this category [27], further elucidated distinct facets:

▪ Challenge Appraisal, viewing stressful situations as potential achievements.
▪ Centrality Appraisal, which delves into the responsibilities, goals, values, and commitments crucial for assessing events, often resulting in heightened stress.
▪ Threat Appraisal, focusing on the possible harm or loss related to a specific situation.

Following this is the secondary appraisal, characterized by Lazarus and Folkman [22], which delves into an individual’s assessment of resources and strategies available to navigate the stressor. This appraisal examines the perceived capacities, both internally and externally, to tackle stressful circumstances. Aspects like Self-control Appraisal reflect one’s perceived abilities, while Control-Others Appraisal evaluates the support that others might provide. On the other end of the spectrum is Uncontrollability Appraisal, recognizing situations where the stressor seems insurmountable, either by oneself or with external help.

### 2.4. Stress Appraisal among Teachers

Within the demanding landscape of the educational sector, teachers are constantly exposed to myriad stressors, from pedagogical challenges to administrative burdens, interpersonal dynamics, and societal expectations. Navigating this complex terrain requires constant cognitive appraisals, shaping their stress perceptions and coping responses.

Research by Skaalvik and Skaalvik [28] explored the primary appraisals of teachers, noting that many teachers often view challenges, especially in the realm of student engagement and pedagogical innovations, as opportunities for growth and development. However, when faced with administrative hurdles or lack of resources, the centrality appraisal gets accentuated, resulting in heightened stress, as the core values and commitments of teachers come into play.

Moreover, threat appraisal is especially pronounced in regions or institutions undergoing significant changes or facing socio-political disturbances. A study by Yasmeen and Kausar [29] on teachers in challenging socio-political contexts highlighted that teachers frequently appraise situations as threats, especially when safety, job security, or pedagogical freedom is at stake.

The secondary appraisal among teachers often revolves around their perceived efficacy and the support mechanisms in place. For instance, a study underscored how teachers with robust professional development opportunities and supportive institutional cultures often exhibited stronger self-control appraisals [30]. In contrast, those in unsupportive environments or facing resource crunches leaned towards uncontrollability appraisals.

Stress appraisal among teachers, shaped by the unique dynamics of the educational ecosystem, is pivotal in understanding their well-being, performance, and overall job satisfaction. Recognizing the nuances of these appraisals can provide insights into creating supportive environments and interventions tailored to teachers’ needs.

### 2.5. Coping Strategies

In the intricate dance between stressors and human responses lies the realm of coping strategies. These represent the myriad ways in which individuals navigate and respond to taxing scenarios, both internal and external. Lazarus [8] illuminates coping as “*ongoing cognitive and behavioral efforts to manage specific external and or internal demands that are appraised as taxing or exceeding the resources of the person*”. Essentially, coping strategies, as articulated by Folkman and Lazarus [22], are the thoughts and actions employed by individuals to modify their perception of a stressful event and to manage, mitigate, or endure the stress it produces.

Various coping strategies have been delineated over the years; coping caters to different facets of stressors and individual preferences to deal with it [14,31]. Coping is particularly associated with cognitive thinking and manifests itself in various forms [32]. For instance, Problem-Focused Coping involves addressing the root cause of the stressor, essentially trying to solve the problem [33]. Conversely, Emotion-Focused Coping prioritizes regulating emotional responses arising from the stressor, rather than the stressor itself. Some individuals lean towards Avoidance-based Coping, wherein they distance themselves physically or psychologically from the stressor [22]. On the other hand, Approach-based Coping embodies a proactive engagement with the stressor, attempting to manage it directly.

In the intricate landscape of human coping mechanisms, strategies like seeking meaning, social support, or even resorting to humor have been recognized [23]. Whether it is reinterpreting a situation positively through Positive Reframing, seeking solace in religious beliefs, or simply venting to release pent-up emotions, individuals exhibit a diverse range of coping techniques, shaped by their experiences, personalities, and cultural backgrounds.

### 2.6. Coping among Teachers

The educational arena, with its multifaceted challenges, requires teachers to be adept at deploying a range of coping strategies. Teachers, continually navigating pedagogical demands, administrative tasks, and emotional labor, often resort to a blend of coping mechanisms tailored to their unique challenges [28].

Recent literature has delved deep into the coping arsenal of teachers. For instance, a study by Harmsen et al. [10] revealed that many teachers frequently employed Problem-Focused Coping strategies, especially when dealing with pedagogical challenges or curriculum adaptations. When faced with interpersonal conflicts or emotional challenges, many leaned towards Emotion-Focused Coping, trying to regulate their emotional responses.

In the backdrop of increasing administrative pressures and changes in educational landscapes, Seeking Social Support has emerged as a pivotal coping mechanism for teachers [34]. This resonates with the findings of Liang et al. [35] and Chong and Chan [36], who underscored the significance of organizational and peer support and mentorship programs in bolstering teachers’ coping capacities.

Many coping strategies which are mostly used by Pakistani teachers are presented in the study, which include time management, eating habits, offer prayers, spending quality time with friends and family members, reading different books, writing their thoughts, multiple exercises, yoga and meditation, workshops related to stress management and medication [37]. Interestingly, many teachers also employ Meaning-focused coping, attempting to derive a deeper purpose or significance from their roles and challenges, especially in contexts where they deal with diverse classrooms or socio-economically disadvantaged students [34].

Based on the above literature review, it can be stated that teachers, given their pivotal roles and the unique stressors they face, exhibit a dynamic range of coping strategies. Recognizing and nurturing these mechanisms can have profound implications for their well-being, efficacy, and the broader educational ecosystem.

## 3. Purpose and Hypotheses

### 3.1. Purpose

The modern era, characterized by rapid societal changes, technological advancements, and evolving job dynamics, has posed unique challenges to professionals, including teachers. The multifaceted roles of college teachers, influenced by various external and internal factors, have made them particularly susceptible to perceived stress and varied stress appraisals. Coping strategies, a natural sequel to these appraisals, also vary based on numerous individual and situational determinants. Given this context, it becomes essential to delve deep into the intricate interplay between perceived stress, stress appraisal, and coping strategies among college teachers. Additionally, gaining knowledge about how job-related factors like gender, marital status, and institutional affiliation impact these aspects can offer understanding for developing policies, establishing support systems in educational institutions, and promoting individual well-being. Consequently, this research aims to explore the connections between perceived stress, stress appraisal, and associated level of coping while also examining the effects of gender, marital status, and institutional affiliation on these constructs within the context of college teachers.

### 3.2. Development of Hypotheses

The formulation of the study’s hypotheses is rooted in a synthesis of the existing literature on perceived stress, stress appraisal, and coping strategies, with a special focus on teachers.

Relationship Between Constructs: Research has consistently highlighted that the way individuals perceive stress has a direct bearing on their appraisal of such stress and the level of coping they employ [6]. In the context of college teachers, given their unique challenges, understanding this association can provide insights into their psychological well-being and professional efficacy.

Influence of Gender: Gender’s influence on stress and coping is not just biologically determined but is deeply embedded within societal constructs and expectations. Men and women, owing to societal roles and cultural nuances, particularly in regions like Pakistan, might perceive and deal with stressors distinctly [18]. For instance, in certain cultural contexts, women teachers might face additional pressures related to work–life balance, societal expectations, and professional growth.

Impact of Marital Status: An individual’s marital status plays a crucial role in their stress perception and coping mechanisms. Marriage often brings along added responsibilities and commitments, potentially amplifying stressors or altering coping mechanisms. Conversely, unmarried individuals might face societal pressures or feelings of isolation, which can influence their stress appraisal and coping strategies [16].

Effect of Institutional Affiliation: The dichotomy between private and government institutions significantly affects the stress landscape for teachers. For instance, private institutions in regions like Pakistan often come with challenges like lack of job security and comparatively lower remuneration, potentially escalating perceived stress among teachers [3,19]. These institutional discrepancies inherently influence the type and magnitude of stressors, the appraisal of such stressors, and the consequent coping mechanisms teachers resort to [3].

Given the aforementioned rationale, the hypotheses for the study are as follows:

**H1.**  *There is a significant association between Perceived Stress, Stress Appraisal, and Coping strategies among College Teachers.*

**H2.**  *Gender significantly influences Perceived Stress, Stress Appraisal, and Coping strategies among College Teachers.*

**H3.**  *Marital status significantly affects Perceived Stress, Stress Appraisal, and Coping strategies among College Teachers.*

**H4.**  *College Teachers’ Perceived Stress, Stress Appraisal, and Coping strategies significantly vary based on Institutional Affiliation (Private vs. Government).*

## 4. Measures and Methods

### 4.1. Participant’s Information

The study comprised a diverse group of 300 college teachers with a response rate of 68%, hailing from various parts of Pakistan, sampled using a non-random convenient sampling procedure. This cohort was balanced in terms of gender, with 150 men participants and 150 women participants. The age range of the participants spanned between 25 to 55 years, ensuring a comprehensive representation of early, mid-, and late-career teachers. All participants had been working as college teachers for a minimum duration of six months, ensuring a basic familiarity and engagement with the educational environment. The sample was extracted from a range of educational institutions, encompassing private, semi-government, and government colleges, providing a panoramic view of the diverse institutional cultures and dynamics.

### 4.2. Data Collection Procedure

To ensure the accuracy, consistency, and integrity of the data collection process, the following methodology was followed:

Before starting the data collection, permission and consent was obtained from the respective college administrations, and clearance was sought from the Federal Education Directorate to ensure that this study aligns with the ethical standards. The data collection took place during working hours to minimize disruption to participants’ routines and create an environment for them to provide authentic responses.

Before distributing the questionnaires, a session was held with the participants of the survey. During this session, the objectives of the study were clarified to them and assured them that strict confidentiality protocols will be in place. Participants were informed that their personal information would remain anonymous as no personal identities were collected. Their responses would be solely used for research purposes without any identifiable connection to them.

Moreover, instead of having participants of the survey independently complete the questionnaires, the researcher took an interactive approach to prevent any unanswered items or misunderstandings. Participants verbally shared their responses while the researcher recorded them on their questionnaire sheets. This method was adopted to ensure clarity, address any ambiguities in real time, and enhance the accuracy of the responses. It also created a platform for participants to seek immediate clarifications on any item they found ambiguous. Once filled, each questionnaire was immediately placed in an envelope, reinforcing the commitment to participant anonymity. No identifiable markers or labels were placed on the envelopes, and participants were reminded that their responses would be aggregated in the final analysis, further ensuring the absence of individual identification.

### 4.3. Measures Used for the Research

#### 4.3.1. Perceived Stress Scale (PSS)

Developed by Cohen, Kamarck, and Mermelstein [38], the PSS is a widely recognized instrument designed to measure the degree to which situations in one’s life are perceived as stressful. The scale does not focus on specific events but rather on how individuals perceive their lives and how unpredictable, uncontrollable, and overloaded they find them to be. Given the multifaceted challenges college teachers face, from pedagogical pressures to institutional dynamics and personal life balances, the PSS provides a comprehensive view of their overall perceived stress levels and also with respect to sub-dimensions, e.g., vocational strain, psychological strain, interpersonal strain, and physical strain.

All questions on the 40-item PSS scale are structured on a 5-point Likert scale, allowing responses to range from never (0) to very often (4). The cumulative score of the four-factor model spans from 0 to 160, with higher scores indicating elevated levels of perceived stress. In the context of Pakistan, the PSS has demonstrated commendable reliability. Zamaan and Ali [39] reported a reliability coefficient of >0.78 when assessing the scale’s psychometric properties within the Pakistani populace. This finding aligns with another study by Tariq et al. [40] that reported an even higher reliability coefficient of 0.85. Such robust reliability metrics underscore the scale’s suitability for capturing the intricacies of perceived stress among college teachers in Pakistan.

#### 4.3.2. Stress Appraisal Measure (SAM)

Developed by Peacock and Wong [41], SAM is a comprehensive tool to measure an individual’s appraisal of stress. It disentangles the primary appraisal into its constituents like Threat, Challenge, and Centrality. Threat appraisals involve the potential for hard loss in the future, and challenge appraisals reflect the anticipation of gain or growth from the experience. Centrality refers to the perceived importance of an event for one’s well-being. Simultaneously, it delves into secondary appraisal, discerning nuances on perceptions of controllability of situation by Self-control, Control by others, and Uncontrollability. Based on the cognitive–relational theory, the tool provides a detailed view of how participants perceive and evaluate stressors. SAM has 28 items with possible responses from 1 to 5 and a maximum score of 140 [41].

Given that college teachers in Pakistan grapple with a variety of stressors, from institutional pressures to societal expectations, the SAM allows for a detailed understanding of their initial reactions (primary appraisal) and subsequent assessments (secondary appraisal) of these challenges. The stress appraisal measure has Cronbach’s alpha value of 0.95 for Pakistan [42].

#### 4.3.3. Brief COPE

Developed by Charles S. Carver [43], Brief COPE is an instrument tailored to measure coping orientation to problems experienced with coping strategies employed by individuals when faced with stressors. Broadly, the total score of the measure provides an estimate of an effort used to minimize distress associated with negative life experiences. It canvases a range of coping strategies from problem-focused approaches to emotion-centric and avoidant ones. With subscales like Self-distraction, Denial, Substance Use, Behavioural disengagement, Emotional Support, Venting, Humour, Acceptance, Self-Blame, Religion, Active Coping, Use of Instrumental Support, Positive Reframing, and Planning, it offers a holistic view of a participant’s coping arsenal [43].

There are 28 items in the Brief COPE, with a Likert scale from 1 to 4 having choices 1 = I haven’t been doing this at all, to 4 = I’ve been doing this a lot. The overall score can be up to 112. No items are reverse-scored. The Brief COPE has good test–retest reliability, with a coefficient ranging from 0.50 to 0.90 [43].

Given the diverse challenges teachers face, they are likely to employ a spectrum of coping strategies, and this tool is adept at capturing that diversity.

The selected measures, with their comprehensive frameworks, cultural relevance, and domain-specific resonances, are ideally poised to provide a deep, nuanced, and holistic understanding of the perceived stress, stress appraisal, and coping strategies among college teachers in Pakistan.

### 4.4. Statistical Analysis

Given the multifaceted nature of the study and its objectives, a combination of statistical tests was employed to discern patterns and relationships:

Pearson Correlation Analysis: This was performed to establish the strength and direction of the linear relationship between Perceived Stress, Stress Appraisal, and Coping Strategies. It provided insights into how these primary constructs interacted with each other.

*T*-tests: Given the bifurcation of the sample based on categorical variables like Gender (Men/Women), Marital Status (Married/Unmarried), and Institutional Affiliation (Private/Government), independent sample *t*-tests were conducted. These tests facilitated comparisons between groups, highlighting if there were significant differences in their Perceived Stress, Stress Appraisal, and Coping Strategies based on these demographic and professional categorizations.

All analyses were conducted using a 0.05 significance level, and data were processed using SPSS version 28.

## 5. Analysis and Results

### 5.1. Descriptive Statistics of the Sample

The subsequent section describes the findings derived from the rigorous analysis of the data collected from the sample of college teachers in Pakistan. By employing a suite of statistical tools, the study sought to explore the intricate relationships between perceived stress, stress appraisal, and coping strategies, while also discerning the potential influence of demographic and occupational variables like gender, marital status, and institutional affiliation. The results provide a comprehensive snapshot, shedding light on the unique experiences, perceptions, and strategies of teachers within the specific cultural and institutional context of Pakistan. Let us delve into the specific outcomes and their implications.

Frequency Table 1 of various Demographic variables, first of all frequency of different demographics (age, Gender, Institutional affiliation, marital status) variables have been measured to get clear picture, frequencies and percentages on each demographic variable have been calculated in following table.

Descriptive statistics were computed to provide an overview of the sample demographics. Out of the 300 participants, 223 (74.3%) fell within the age range of 25 to 40 years, while the remaining 77 (25.7%) were aged between 41 and 55 years. The sample maintained a balanced gender distribution, with 150 (50.0%) men and an equal number of women. When examining institutional affiliations, 173 participants (57.7%) were associated with government colleges, contrasting with the 127 (42.3%) representing private institutions. In terms of marital status, 165 (55.0%) participants identified as single or unmarried, whereas the other 135 (45.0%) were married.

### 5.2. Reliability Analysis of the Instruments

To determine the reliability and internal consistency of the instruments used in this study, the Cronbach Alpha Coefficient was employed.

#### 5.2.1. Perceived Strain Questionnaire (PSQ)

The overall reliability for the 40 items of PSQ was excellent, registering a Cronbach Alpha of 0.96.

#### 5.2.2. Stress Appraisal Measure (SAM)

As a whole, the 28 items of SAM exhibited an outstanding Cronbach Alpha of 0.94, underscoring its excellent consistency. On examining its subscales, the coefficients spanned a range between 0.62 and 0.82. While the lower bound of this range suggests moderate reliability, the upper bound, nearing 0.82, denotes robust internal consistency for certain subscales.

#### 5.2.3. Brief COPE

The 28 items of Brief COPE have shown that a Cronbach Alpha of 0.91, a score lying in the excellent spectrum, affirms the instrument’s reliability and suitability for the sample. The chosen instruments exhibit commendable-to-excellent reliability coefficients, confirming their appropriateness for capturing the nuances of stress, appraisal, and coping strategies within the specified cohort.

Table 2 Correlation Analysis of PSQ and SAM: To delve into the interplay between Perceived Stress and Stress Appraisal among college teachers, a statistical exploration was undertaken using Pearson correlation coefficient tests. These tests were tailored to discern the strength and direction of relationships among variables gauged by their designated scales: The Personal Strain Questionnaire (PSQ) for Perceived Stress, the Stress Appraisal Measure (SAM) for Stress Appraisal [32].

From the data presented in Table 2, a notable relationship emerges between Perceived Stress, as captured by PSQ, and Stress Appraisal, gauged by SAM. The Pearson correlation value, r = 0.353 **, implies a moderate positive relationship. The correlation coefficient between Vocational Strain subscale values is also significant, with total of appraisal being significant (r = 0.334 **). On the basis of results, we can infer that the perception of stress is related with cognitive appraisal of stress. The relationship between psychological strain and stress appraisal is (r = 0.338 **) significant. Interpersonal strain with correlation coefficient (r = 0.330 **) and physical strain relationship (r = 0.310 **) is significant with stress appraisal measure. The significance level, *p* < 0.001, accentuates the robustness of this relationship, suggesting that as perceived stress increases, the stress appraisal by teachers also escalates.

Similarly, Table 3 brings to light a significant relationship between the Personal Strain Questionnaire (via PSQ) and Coping Strategies (via Brief COPE). The correlation coefficient, r = 0.456 **, suggests a more pronounced positive association compared to the previous correlation coefficient between subscales of coping that are also significant with the total of PSQ. But the subscale behavioral disengagement of coping was non-significant with interpersonal strain (r = 0.840, *p* > 0.05). On the basis of the overall results, it seems true that stress is correlated with coping. Again, with a significance value of *p* < 0.05, it is evident that heightened perceived stress aligns with specific coping strategies adopted by the teachers.

Drawing from the insights presented in Table 2 and Table 3, there is empirical support for Hypothesis H1. It postulates a significant interconnectedness among Perceived Stress, Stress Appraisal, and Coping Strategies within the realm of college teachers. The data solidly reaffirms this hypothesis, emphasizing the intricate web of stress perception, its evaluation, and the coping mechanisms teachers employ in their professional environment.

### 5.3. Analyzing Relationship of Gender, Marital Status, and Institutional Affiliation with Stress, Appraisal, and Coping Strategies

In social and behavioral research, understanding demographic nuances, such as gender differences, is imperative, especially when investigating perceptions and behaviors. In the realm of stress, appraisal, and coping, gender nuances might be influenced by a confluence of societal expectations, biological factors, and personal experiences.

To dissect the impact of gender on these parameters, independent samples *t*-tests were employed. The *t*-test is ideal for comparing means between two independent groups, in this case, men and women. Given our sample’s natural grouping of equal men and women participants (150 each), the *t*-test offers a robust methodology to elucidate potential differences.

For total PSQ, men registered an average score of mean 118.58, suggesting that they perceive moderately high levels of stress. Women, on the other hand, scored slightly higher with an average of 126.69. The t-value of 4.54 with a significant *p*-value of 0.001 indicates a statistically significant difference between the stress perceptions of men and women teachers. The results highlight a notable gender disparity, with women perceiving heightened levels of stress compared to men.

For total SAM, men had an average score of 64.07, indicating their general appraisal of stress. While women presented a notably higher mean of 72.53 and a pronounced t-value of 3.50 and a *p*-value of 0.001, the results underscore a significant gender disparity in stress appraisal, with women appraising their stressors more intensely than men.

For the Brief COPE, men depicted an average coping score of 79.59, suggesting their general coping strategies. Women reported a slightly lower mean score of 74.29 with a t-value of 3.52, paired with a significant *p*-value of 0.001, revealing a discernible gender difference in coping strategies, although with a twist. Men tend to employ coping strategies more compared to women, suggesting potential gender-specific coping mechanisms or perhaps varied stressors that each gender encounters.

Men tend to report higher levels of humor, acceptance, and religion, while women tend to report higher levels of self-blame. Subscales also show statistically significant mean differences between the two groups (*p* < 0.05). There are significant differences between the two groups in various coping strategies measured by the Brief COPE scale. The men tend to utilize more adaptive coping strategies such as self-distraction Men (mean = 6.45, SD = 2.22), Women (mean = 5.74, SD = 1.95), active coping Men (mean = 5.32, SD = 1.68), Women (mean = 4.62, SD = 1.49), seeking instrumental support Men (mean = 5.68, SD = 1.42), Women (mean = 4.67, SD = 1.47), humor, acceptance, and religion, while the second group tends to exhibit more maladaptive coping strategies like self-blame.

Drawing from the results outlined in Table 4 and Table 5, there is ample empirical validation to assert that Hypothesis H2 is supported by the data. Gender, as a variable, plays a crucial role in how stress is perceived, appraised, and coped with among college teachers. The results from Table 4 unequivocally substantiate this, reinforcing the essence of H2 and highlighting the need for gender-specific considerations in interventions or institutional policies tailored for teachers.

Understanding the relationship between marital status and individual response to stress in an academic setting is imperative for holistic faculty welfare. The participants for this analysis were distinctly grouped into two: 135 married teachers and a slightly larger group of 165 single teachers.

For perceived stress, the single group averaged a stress score of 112.50, indicating a moderate level of stress perception. Conversely, the married group reported a marginally heightened mean score of 126.0. A t-value of 3.42 and a *p*-value of 0.001 demonstrate a statistically significant difference in stress perception between the two groups, with married teachers reporting slightly heightened levels of stress.

For stress appraisal, the single group registered an average appraisal score of 63.64, suggesting their particular manner of interpreting stressors. The married group, however, posted a mean score of 74.01, indicating a nuanced difference in their appraisal.

The *t*-value of 4.32, combined with a *p*-value of 0.01, points towards a significant variance in stress appraisal contingent on marital status. Married teachers, on average, interpret stressors with greater intensity than their single peers.

For coping strategies, the single teachers reported a mean coping score of 72.41. In contrast, the married teachers exhibited a mean score of 77.70. With a notable t-value of 3.24 and a *p*-value of 0.001, it is clear that marital status undeniably molds the coping strategies teachers employ. Married teachers appear to harness slightly varied or increased coping strategies compared to those who are single.

Marital status has an impact on certain coping strategies. Married individuals tend to report higher levels of active coping in the married group (mean = 5.10, SD = 1.59) compared to singles (mean = 4.87, SD = 1.65). For venting, married individuals are scoring slightly lower (mean = 5.27, SD = 1.56) compared to singles (mean = 5.10, SD = 1.50). However, marital status does not seem to significantly influence other coping strategies measured by the Brief COPE scale.

The insightful results from Table 6 and Table 7 offer substantial empirical support for Hypothesis H3. The data illuminates the pronounced influence marital status exerts on stress perception, its ensuing appraisal, and the resultant coping mechanisms. In the academic realm of college teachers, marital status emerges not just as a demographic detail but as a significant predictor of stress dynamics. This underscores the need for tailored well-being programs and understanding that address the unique stress experiences and needs of both married and unmarried faculty members.

The data in Table 8 provide a holistic view of the relationship of institutional affiliation (teachers of different Government vs. Private institutions) with Stress, Appraisal and Coping Strategies.

For this purpose, the sample was divided into two groups: 129 teachers from private colleges and 171 from government colleges. From the results, it can be stated that teachers in private colleges had an average stress score of 124.40, while teachers in government colleges scored slightly lower at 107.60. The significant difference (*p*-value of 0.001) suggests that teachers from private institutions feel a bit more stressed compared to those in government settings.

Also, for stress appraisal, on average, private college teachers had a score of 71.61, indicating how they perceive and evaluate stress. Government college teachers scored 64.15 on average. The big difference in scores (*p*-value of 0.001) shows that teachers in private colleges, perhaps confronting fluid organizational mandates or heightened performance pressures, seem to evaluate and interpret stressors with greater intensity.

When it comes to coping strategies, the analysis found that private college teachers had an average coping score of 77.5, while government college teachers had an average score of 72.9. The significant difference (*p*-value of 0.01) means that teachers in these two settings use slightly different strategies to handle their stress.

Results suggest that institutional affiliation (Private vs. Government) has an impact on various coping strategies measured by the Brief COPE scale. Individuals affiliated with government institutions tend to exhibit higher levels of coping strategies such as active coping private (P): mean = 4.84, SD = 1.64 government (G): mean = 5.06, SD = 1.61, venting private (mean = 5.56, SD = 1.53) G: (mean = 5.84, SD = 1.47), humor, acceptance, religion, self-distraction, denial, substance use, emotional support, instrumental support, behavioral disengagement, personal reframing, and planning compared to those affiliated with private institutions. These subscales show statistically significant mean differences between private and government-affiliated individuals (*p* < 0.05).

The data in Table 8 and Table 9 confirm hypothesis H4, proving that the kind of institution, whether private or governmental, does play a role in how teachers experience stress, how they view it, and how they deal with it. This finding is essential as it can guide institutions in providing the right kind of support and resources to their teachers based on their unique challenges.

The relationships between perceived stress, stress appraisal, and coping strategies among college teachers are significantly influenced by their gender, marital status, and institutional affiliation. The data underscore the multidimensional nature of stress and coping in the academic environment, revealing that individual and institutional factors intertwine in shaping the experiences of teachers. The nuances in stress perception and coping mechanisms among different subgroups highlight the need for tailored interventions and support. As we transition to discussing these findings in the broader context of the existing literature, it becomes clear that understanding these variations is essential in crafting meaningful, targeted solutions to address the challenges faced by teachers.

## 6. Discussion

In our quest to understand the multifaceted dimensions of stress, its appraisal, and the coping strategies employed by college teachers, this discussion section aims to weave together the empirical findings with established theoretical frameworks and prior research narratives. By juxtaposing our observations with the broader academic discourse, we aspire to provide a comprehensive and contextual understanding of the dynamics at play, especially within the unique socio-cultural fabric of the Pakistani educational sector.

The analysis in this research has underpinned the dynamic interplay among perceived stress, stress appraisal, and coping strategies, which was articulated through correlation associated with stressors. The profound positive relationship between perceived stress and stress appraisal reaffirms that as teachers encounter more pronounced stressors, their interpretation and evaluation of these stressors intensify. Further, as stress amplifies, teachers seem to adapt and alter their coping strategies. This overarching dynamic presents a coherent narrative resonating with established theories and findings in the domain and supporting the formulated hypothesis (H1).

At the foundation of our understanding of stress lies the seminal work of Lazarus. In his exposition, stress is not merely an external stimulus or an internal response. Instead, Lazarus framed stress as a nuanced interaction between the individual and the environment, modulated by personal interpretations and the perceived adequacy of coping resources. In our study, the correlation between perceived stress and stress appraisal validates this perspective, underscoring that stress is not merely about external triggers but how these triggers are appraised in light of available coping mechanisms [22].

These results also showed that employed coping strategies could be best understood if the boundaries for problem-focused coping and emotion-focused coping are not so rigid. Because both types of coping domains, namely problem-focused and emotional-focused coping, tend to employ some part of those strategies which are theoretically not considered in that domain [44]. In one study, Pakenham and Rinaldis [45] found that healthy adjustments for individuals suffering from AIDS could be predicted by the cognitive appraisal of challenge and controllability. Also due to the dispositional traits of individuals, the same type of strategy is utilized at first, as part of problem-focused coping, but due to persistent stressors, these are directed towards emotion-focused coping [46].

### 6.1. Gender-Based Differences in Perceived Stress, Stress Appraisal, and Coping Strategies

The findings of this study underscore distinct gender-based discrepancies in perceived stress, stress appraisal, and coping strategies among college teachers. In alignment with the hypothesis (H2), the results unequivocally indicate that gender significantly influences these dimensions among teachers. Notably, women reported heightened stress levels and a more profound appraisal of stressors compared to their counterparts. These findings resonate with the broader academic narrative, thereby bolstering the understanding that gender intricately interweaves with stress perception and appraisal.

The observed differences can be contextualized within the socio-cultural fabric of Pakistan. The country’s patriarchal culture, which traditionally accords a dominant role to men and often limits women’s professional engagements, can amplify stress levels for women. In such a society, women teachers might grapple with dual pressures—the intrinsic challenges of their profession compounded by societal expectations and constraints. This could potentially elucidate the elevated stress levels and coping mechanisms discerned among women teachers in our sample.

Graves et al. [18] further enrich this narrative by highlighting that women showcased higher stress levels and predominantly employ emotion-focused coping and adopt strategies such as self-distraction, emotional support seeking, and venting more than men. Such patterns, as mirrored in our study, offer deeper insights into the gender-specific coping strategies amidst stressors.

Moreover, the insights from Antoniou et al. [47] suggest that women teachers confront intensified stress, particularly concerning student interactions, workload, and emotional exhaustion. These nuances, seen in the backdrop of a society with deeply entrenched gender roles, can accentuate the stress dimensions for women teachers in Pakistan.

Interestingly, the findings by Chong and Chan [36] present an outlier in the narrative, suggesting that men and women teachers in Hong Kong experience equivalent stress levels. While this might seem contradictory at first, it underscores the importance of contextual and cultural factors in shaping stress perceptions. This also suggests that while overarching trends are discernible, regional and cultural specificities can significantly modulate these patterns.

However, the broader historical context, as highlighted by Hsu and Barrett [17], reaffirms our findings and the dominant narrative in the literature: women consistently report elevated perceived stress compared to men. Whether this is attributed to inherent biological differences, societal constructs, or a blend of both, warrants deeper exploration.

While this study sheds light on the nuanced interplay between gender and stress dimensions in the context of college teachers, it also underlines the need for further culturally specific research to refine our understanding of these dynamics.

### 6.2. Marital Status and Its Influence on Perceived Stress, Stress Appraisal, and Coping Strategies

The exploration of the interplay between marital status and its bearing on stress perception, appraisal, and coping strategies among teachers is integral for understanding the complete psychological landscape they navigate. This study, through rigorous *t*-tests, has shed light on the nuanced ways in which marital status shapes these constructs among college teachers.

In line with the findings, a notable dichotomy emerges between unmarried and married teachers. Specifically, unmarried teachers exhibited moderate stress levels, whereas their married counterparts reported slightly elevated stress levels. Similarly, the stress appraisal scores between the two groups also presented a divergence, suggesting that marital status may play a role in how teachers interpret and assess stressors. On the coping front, the results hinted at a distinct pattern, with married teachers gravitating toward more intensified coping strategies compared to their unmarried peers. This congruence provides compelling empirical backing to Hypothesis H3.

Delving into the broader literature lends further contextual depth to the findings. Ghafoor et al. [16] shed light on the pronounced stress levels amongst unmarried students in areas related to career prospects and academic obligations. Although this study zeroes in on students, the parallels to our observations regarding unmarried teachers are hard to miss. In the academic sphere, whether student or teacher, being unmarried seems to magnify certain stressors.

Contrastingly, Hsu and Barrett [17] posited that certain unmarried individuals, particularly women, might exhibit better stress and coping profiles than those continuously married. This somewhat counterintuitive finding underscores the complex mosaic of factors, including gender, that interplay with marital status to shape stress dynamics. Our observations, especially regarding unmarried teachers’ stress appraisal, resonate with this nuanced understanding.

Ta et al. [20] further enriches this narrative by emphasizing the intricate relationship between singlehood and perceived stress, especially in domains like social engagements, loneliness, and financial constraints. Their findings lend credence to the idea that marital status can be a pivotal determinant of exposure to specific stressors, consequently influencing mental well-being.

When contextualized within the cultural backdrop of Pakistan, a society with deeply entrenched familial and societal expectations, it is conceivable that marital status exerts pronounced effects on teachers’ stress perceptions and coping strategies. Marriage in Pakistan is not just a personal union; it is intricately woven with extended family dynamics, societal expectations, and cultural obligations. These additional layers could potentially amplify the stressors faced by married teachers, as seen in our findings.

While this study carves out a clearer understanding of the relationship between marital status and stress dimensions among college teachers, it also underscores the importance of considering societal and cultural facets in this interplay. The confluence of these insights paints a more comprehensive picture of the academic stress landscape navigated by teachers in Pakistan.

### 6.3. Impact of Institutional Affiliation on Perceived Stress, Stress Appraisal, and Coping Strategies

In the academic realm, the institutional environment, with its unique demands, expectations, and operational idiosyncrasies, can profoundly mold the psychological experiences of teachers. The crux of this research centered around understanding how the institutional affiliations Private vs. Government affect stress dynamics among college teachers.

The statistical delineation revealed some compelling insights. Private college teachers exhibited heightened stress levels, more intense stress appraisal, and slightly altered coping strategies compared to their governmental counterparts. This difference, particularly in stress perception and appraisal, might stem from the distinct challenges intrinsic to each institutional setting.

This study’s outcomes seem to resonate with the broader socio-cultural landscape of Pakistan’s educational sector. Historically, private institutions in the country, while offering a degree of operational flexibility, often grapple with resource constraints, lower remunerations, and job insecurity. This could significantly increase stress levels, corroborating the findings that private college teachers perceive and appraise stress more intensely.

Previously, Griffit [48] explained that older and more experienced teachers perceive less stress as compare to non-experienced teachers or newcomers. He also reported that junior teachers are considered more stressed as compared to senior faculty.

Yet, juxtaposing the results with the existing literature illuminates intriguing contrasts. Siddiqui [19] posited that public school teachers, contrary to our findings, bore the brunt of heightened occupational stress. This discrepancy can be attributed to the evolving dynamics of the education sector, where, over time, private institutions might be grappling with intensifying challenges, thereby escalating teacher stress levels. Furthermore, the overarching socio-economic changes and the rising expectations in the private sector might be exerting additional pressures on teachers.

Another intriguing observation came from Ali and Kumar [3], which underscored parity in stress levels between teachers of private and government schools. While initially seeming at odds with our findings, it is pivotal to understand the regional or demographic specificities of their study cohort. Moreover, the dynamics of school-level education and college-level instruction can vary significantly, thus presenting divergent stress landscapes.

Lastly, the global context offers further nuance. Contrary to Pakistan, where private institutions might grapple with financial constraints and limited resources, the Western world often sees private institutions flush with resources and infrastructure. This stark contrast in institutional dynamics between regions underscores the significance of geo-specific research to understand the intricate fabric of academic stress.

Our findings, while aligning with the prevailing trends in Pakistan’s educational sector, also spotlight the intricate interplay of institutional dynamics, socio-cultural contexts, and individual psychology. Such insights are pivotal for shaping institutional policies, fostering faculty welfare, and optimizing the educational ecosystem.

In retrospect, the analysis and exploration has unraveled intricate relationships, subtle nuances, and distinct patterns that characterize the stress landscape among college teachers. Drawing parallels with seminal works and recent studies has enriched our understanding and provided a robust context for the findings. As this discussion has been concluded, it becomes evident that the realms of stress perception, appraisal, and coping are inexorably intertwined, demanding continuous exploration and understanding. Future studies, building on this foundation, have the potential to uncover deeper insights and craft strategies that can better support teachers in their pivotal role in society.

## 7. Limitations of the Study

This study provides insights into the stress dynamics, perceptions, and coping strategies of college teachers, though its findings are specifically tied to Pakistan’s socio-cultural and educational context. As such, applying these results to other regions should be carried out with caution due to varying cultural norms and educational systems. However, the similarities in socio-cultural environments and patriarchal structures in many countries suggest that these findings could inform further research in similar settings. Additionally, readers from diverse social backgrounds can gain valuable perspectives on the differences in university environments, highlighting both unique and universal aspects of the academic experience.

Furthermore, due to the cross-sectional nature of this study, it only provides a snapshot of participants’ experiences at a specific point in time. Consequently, it may not fully capture the evolving nature of stress and how it changes with time and the adopting of coping strategies over extended periods. Moreover, there is a possibility that despite the efforts to ensure diversity and representativeness in the sample, certain subgroups, within the teaching community have not had their unique experiences and challenges fully accounted for in this study.

Collectively, while these limitations do not detract from the study’s contributions, they highlight areas for potential refinement in future research endeavors.

## 8. Implications for Practice

The findings of this research offer a wealth of practical insights for educational institutions, policymakers, and teachers themselves. As stress and its subsequent appraisal are proven to be significant factors influencing the well-being and productivity of college teachers, institutions should prioritize the creation of supportive environments. Recognizing the heightened stress levels among women teachers, especially in the context of a patriarchal society like Pakistan, institutions should look into offering gender-specific support programs or workshops, which can help address unique stressors faced by women teachers.

Given the differences in stress perceptions and coping strategies based on institutional affiliations, there is a clear directive for tailored interventions. Private institutions, in particular, should be cognizant of the increased stress their teachers face, potentially due to factors like job insecurity, longer working hours, or reduced remuneration. They might consider adopting measures such as reduced workloads, improved job security, or providing periodic stress management training.

Moreover, understanding that marital status also affects stress perceptions can guide institutional welfare programs. Institutions could offer counseling or support groups, especially catering to the specific needs of unmarried or newly married faculty, helping them navigate personal and professional challenges more effectively.

Lastly, the strong correlation between perceived stress and coping underscores the need for continuous professional development programs focusing on effective stress management techniques. By equipping teachers with tools to appraise and address their stressors proactively, institutions can foster a more resilient, satisfied, and effective teaching workforce.

By embracing the insights of this study, educational entities can make strides in ensuring the mental and emotional well-being of their teachers, which, in turn, will reflect in enhanced teaching outcomes and a more harmonious academic environment.

## 9. Conclusions and Future Directions

In dissecting the myriad of factors that contribute to the perceived stress, stress appraisal, and coping mechanisms among college teachers, this study has illuminated some crucial dynamics that shape their professional lives. The evident gender disparities, the significant influence of marital status, and the differing stress levels between private and governmental institutions highlight the intricate interplay of personal and institutional variables in determining teachers’ stress experiences.

At its core, the study underscores the paramount importance of understanding and addressing the unique stressors college teachers face, as these can profoundly impact their well-being, effectiveness, and, ultimately, the quality of education they impart. Institutions, in light of these findings, carry the onus of crafting supportive, responsive environments that cater to the holistic well-being of their teachers.

However, while the study offers rich insights, it is grounded in the socio-cultural backdrop of Pakistan, a factor that limits its generalizability on a global scale. This very limitation, though, paves the way for future research directions. Comparative studies across different cultural and national contexts could offer a more panoramic view of global educator stress dynamics. Additionally, the exploration of other variables, such as years of teaching experience, educational qualifications, or familial responsibilities, could further enrich our understanding.

Furthermore, as the realm of education continues to evolve, with increasing digitalization and shifts in pedagogical strategies, tracking the evolution of educator stressors and coping mechanisms becomes imperative. The onset of phenomena like technostress or challenges related to remote teaching, especially in the post-COVID-19 era, could be promising areas of exploration in the future.

While this research has paved a path towards a deeper comprehension of educator stress in the Pakistani context, it also beckons the academic community to delve deeper, broadening the scope, and continuously updating our understanding in this ever-evolving domain.

## Figures and Tables

**Table 1:** Frequency table of various demographic variables of sample (n = 300).

Variables	Categories	Frequency	Percentage (%)
Age	25–40	223	74.3
	41–55	77	25.7
Gender	Men	150	50.0
	Women	150	50.0
College type	Government	173	57.7
	Private	127	42.3
Marital status	Single	165	55.0
	Married	135	45.0

**Table 2:** Correlation coefficient of Personal Strain Questionnaire (PSQ) with Stress Appraisal Measure (SAM) and its subscales (n = 300).

Personal Strain Questionnaire	Stress Appraisal Measure							
	Threat	Challenge	Centrality	Controlable-by-Self	Controlable-by-Others	Uncontrol Able	Stressfulness	Total SAM
Vocational Strain	0.273 **	0.327 **	0.369 **	0.334 **	0.333 **	0.425 **	0.334 **	0.334 **
Psychological Strain	0.245 **	0.327 **	0.383 **	0.338 **	0.346 **	0.449 **	0.338 **	0.338 **
Interpersonal Strain	0.261 **	0.292 **	0.319 **	0.330 **	0.287 **	0.375 **	0.330 **	0.330 **
Physical Strain	0.261 **	0.291 **	0.356 **	0.310 **	0.322 **	0.428 **	0.310 **	0.310 **
Total PSQ	0.280 **	0.333 **	0.383 **	0.353 **	0.346 **	0.450 **	0.353 **	0.353 **

** *p* = 0.001.

**Table 3:** Correlation coefficient of Personal Strain Questionnaire (PSQ) with Brief COPE and its subscales (n = 300).

Brief COPE	Personal Strain Questionnaire				
	Vocational Strain	Psychological Strain	Interpersonal Strain	Physical Strain	Total PSQ
Self-distraction	0.368 **	0.426 **	0.462 **	0.414 **	0.449 **
Active coping	0.242 **	0.191 **	0.307 **	0.242 **	0.266 **
Denial	0.341 **	0.400 **	0.435 **	0.384 **	0.419 **
Substance	0.193 **	0.224 **	0.064 **	0.199 **	0.181 **
Emotional support	0.185 **	0.220 **	0.094 **	0.195 **	0.185 **
Instrumental support	0.238 **	0.181 **	0.289 **	0.230 **	0.254 **
Behavior disengagement	0.204 **	0.233 **	0.840	0.204 **	0.245 **
Venting	0.253 **	0.203 **	0.291 **	0.245 **	0.268 **
Positive reframing	0.321 **	0.386 **	0.421 **	0.371 **	0.403 **
Planning	0.273 **	0.327 **	0.367 **	0.325 **	0.347 **
Humor	0.313 **	0.250 **	0.321 **	0.303 **	0.320 **
Acceptance	0.325 **	0.328 **	0.345 **	0.327 **	0.347 **
Religion	0.270 **	0.280 **	0.324 **	0.390 **	0.320 **
Self-blame	0.213 **	0.240 **	0.164 **	0.301 **	0.216 **
Total Brief Cope	0.402 **	0.420 **	0.460 **	0.430 **	0.456 **

** *p* < 0.001.

**Table 4:** Analysis of Stress, Appraisal, and Coping for Gender.

Scales	Men (n = 150)		Women (n = 150)		*t* (n = 300)	*p*-Value
	Mean	SD	Mean	SD		
Personal Stress	118.58	33.88	126.69	36.10	4.54	0.00
Stress Appraisal	64.07	18.49	72.53	23.04	3.50	0.00
Brief COPE	79.59	11.80	74.29	14.11	3.52	0.00

**Table 5:** Mean differences between gender for subscales of Brief COPE (n = 300).

Brief Cope	Men (n = 150)		Women (n = 150)		t (n = 300)	*p*-Value
	M	SD	M	SD		
Active coping	5.32	1.68	4.62	1.49	3.80	0.00
Venting	5.48	1.56	5.26	1.68	4.34	0.00
Humor	5.96	1.58	5.45	2.07	2.38	0.01
Acceptance	6.03	1.57	5.47	2.06	2.60	0.01
Religion	6.12	1.71	5.72	1.84	1.94	0.05
Self-blame	5.27	1.75	4.56	1.49	3.75	0.00
Self-distraction	6.45	2.22	5.74	1.95	2.92	0.00
Denial	5.47	2.24	5.88	1.80	1.12	0.00
Substance use	6.43	2.26	1.42	0.98	1.41	0.01
Emotional support	2.38	1.16	4.54	1.94	2.39	0.00
Instrumental support	5.68	1.42	4.67	1.47	6.01	0.00
Behavioral disengagement	3.54	1.24	2.90	1.12	3.43	0.00
Personal reframing	4.22	1.70	4.12	1.67	1.32	0.01
Planning	3.66	1.41	3.21	1.12	2.49	0.00

**Table 6:** Analysis of Stress, Appraisal, and Coping Strategies for different Marital Status.

Scales	Married (n = 135)		Single (n = 165)		*t*-Value	*p*-Value
	Mean	SD	Mean	SD		
Personal Strain	126.01	33.32	112.50	30.80	3.42	0.00
Stress Appraisal	74.01	23.19	63.64	18.36	4.32	0.00
Brief COPE	77.70	13.51	72.41	14.68	3.24	0.00

**Table 7:** Mean differences on marital status for subscales of Brief COPE (n = 300).

Scales	Married (n = 165)		Unmarried (n = 135)		*t*-Value	*p*-Value
	M	SD	M	SD		
Active coping	5.10	1.59	4.87	1.65	3.80	0.01
Venting	5.27	1.56	5.10	1.50	6.01	0.00
Humor	5.90	1.94	5.55	1.77	−1.60	0.11
Acceptance	5.93	1.94	5.61	1.76	−1.48	0.13
Religion	6.27	1.50	6.07	1.66	−1.08	0.01
Self-blame	4.98	1.63	4.86	1.68	−0.606	0.00
Self-distraction	6.38	1.48	5.90	1.95	−2.32	0.01
Denial	6.38	1.45	5.98	1.82	−2.12	0.01
Substance use	2.90	1.26	2.58	0.98	−2.41	0.01
Emotional support	2.38	1.16	2.54	0.94	−2.39	0.01
Instrumental support	5.27	1.56	5.10	1.50	−0.99	0.01
Behavioral disengagement	2.93	1.27	2.59	0.993	−2.53	0.01
Personal reframing	6.41	1.38	5.95	1.87	−2.38	0.01
Planning	6.36	1.53	6.09	1.67	−1.14	0.00

**Table 8:** Analysis of Stress, Appraisal, and Coping Strategies for different Institutions (Government VS. Private).

Scales	Private (n = 129)		Government (n = 171)		*t*-Value	*p*-Value
	Mean	SD	Mean	SD		
Personal Strain	124.40	34.39	107.60	34.46	4.18	0.00
Stress Appraisal	71.61	21.68	64.15	20.65	3.01	0.00
Brief COPE	77.53	13.25	72.95	16.77	2.55	0.01

**Table 9:** Mean difference on Institutional Affiliation for Subscales of Brief COPE (n = 300).

Scales	Private (n = 129)		Government (n = 171)		*t*-Value	*p*-Value
	M	SD	M	SD		
Active coping	4.84	1.64	5.06	1.61	1.161	0.00
Venting	5.56	1.53	5.84	1.47	1.59	0.01
Humor	5.56	1.95	5.82	1.78	1.18	0.00
Acceptance	5.57	1.93	5.88	1.78	1.44	0.00
Religion	6.36	1.40	6.01	1.67	1.92	0.01
Self-blame	4.81	1.66	4.99	1.66	1.91	0.00
Self-distraction	6.20	1.72	6.05	1.80	2.73	0.00
Denial	6.22	1.66	6.11	1.68	S2.56	0.01
Substance use	2.54	0.998	2.86	1.20	2.38	0.00
Emotional support	2.55	0.957	2.76	1.11	1.67	0.05
Instrumental support	5.09	1.56	5.24	1.51	2.79	0.01
Behavioral disengagement	2.54	0.907	2.90	1.26	2.73	0.00
Personal reframing	6.20	1.70	6.13	1.67	2.32	0.05
Planning	6.36	1.48	6.10	1.70	1.39	0.01

## Data Availability

The data presented in this study are not publicly available to maintain the confidentiality and privacy of the people who participated in the study.
